# Supplementary material for: RYBP Sensitizes Cancer Cells to PARP Inhibitors by Regulating ATM Activity
Source: Int J Mol Sci. 2022 Oct 4;23(19):11764. doi: 10.3390/ijms231911764 (PMC9570458; doi:10.3390/ijms231911764)
Supplement: Supplementary file 1 [file ijms-23-11764-s001.zip › ijms-1758052-supplementary.pdf]

Supplementary Figures

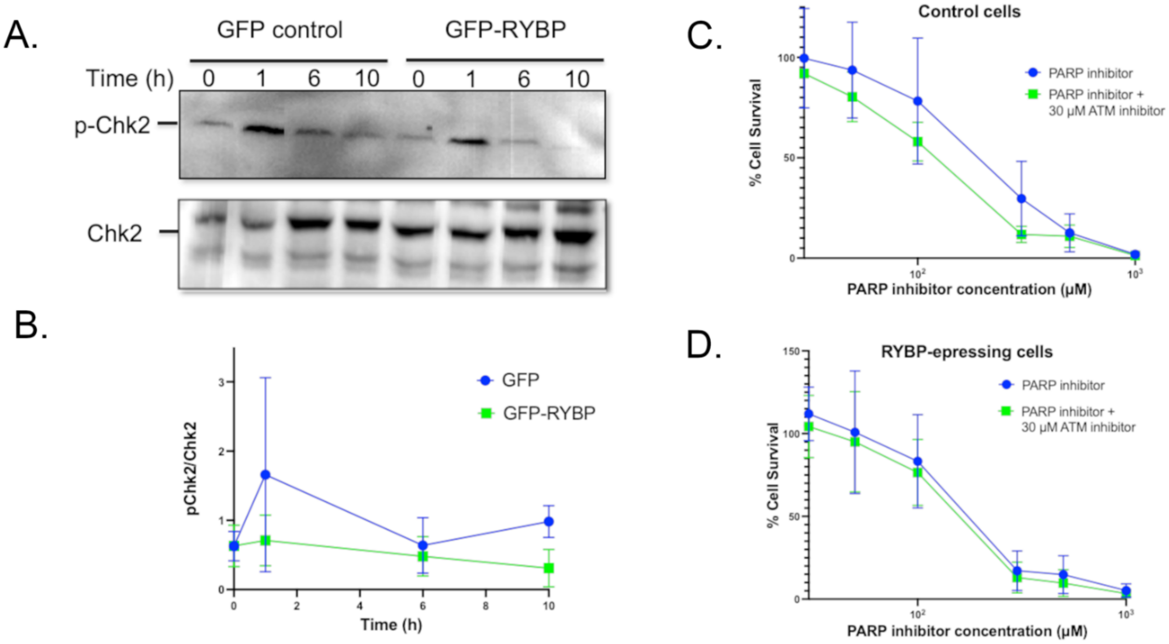

Supplementary Figure S1. Effects of RYBP on ATM activity in SKOV3 ovarian cancer cells.

A. Levels of p-Chk2 and total Chk2 are shown after camptothecin (1 μM) treatment at 0-, 1-, 6- and 10-hour time intervals for GFP-control and GFP-RYBP expressing cells. **B**. Quantification of p-Chk2/Chk2 ratios for GFP-control and GFP-RYBP expressing cells (n = 3 independent experiments). **C.** In control SKOV3 cells, addition of ATM inhibitor (KU-55933) reduced the IC_{50} of PARP inhibitor (ABT-888) from 340 μM to 161 μM. **D**. In RYBP-expressing cells, addition of ATM inhibitor (KU-55933) did not affect the IC_{50} of PARP inhibitor (ABT-888).

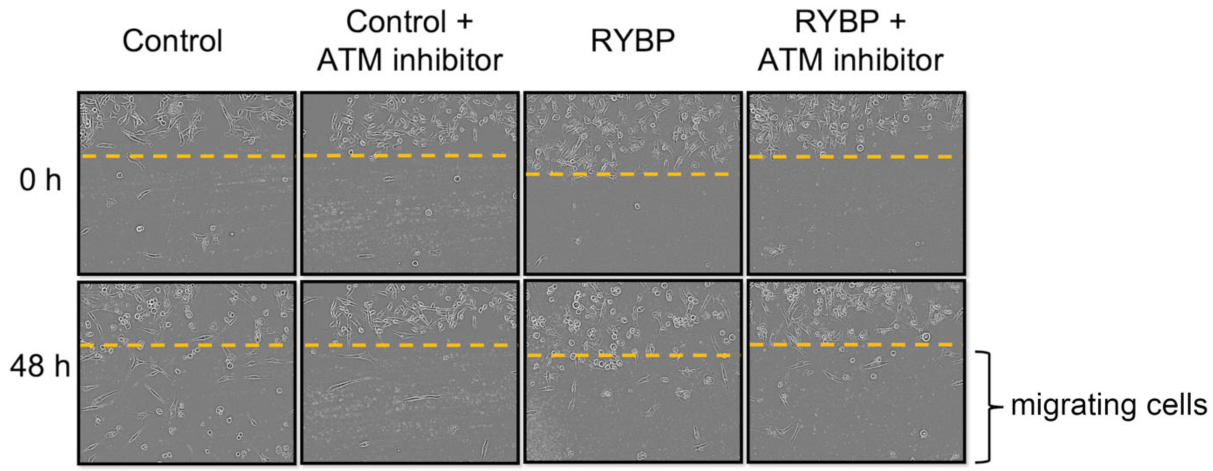

Supplementary Figure S2. **Effects of RYBP and ATM inhibition on MDA-MB-231 cell migration.** The number of MDA-MB-231 migrating cells was reduced by ATM inhibitor or RYBP expression. Combination of RYBP expression and ATM inhibitor did not further reduce cell migration.
